# Surgical treatment of aggressive pituitary adenomas and pituitary carcinomas

**DOI:** 10.1007/s11154-020-09563-8

**Published:** 2020-06-04

**Authors:** Michael Buchfelder, Sven-Martin Schlaffer

**Affiliations:** Department of Neurosurgery, University Hospital Erlangen, Schwabachanlage 6, 91054 Erlangen, Germany

**Keywords:** Invasive adenoma, Aggressive growth, Cavernous sinus, Transsphenoidal surgery, Transcranial surgery, Interdisciplinary management, Pituitary carcinomas

## Abstract

Surgery of aggressive pituitary adenomas and pituitary carcinomas is part of the interdisciplinary management of these difficult to treat tumors. Invasion, giant size and unusual, asymmetric extent of these tumors frequently require modifications or extensions of the standard approaches for transsphenoidal and transcranial surgery. Frequently, only debulking procedures can be performed. In aggressive and hormone secreting adenomas, the remission rates achieved by surgery alone are relatively poor and adjuvant medical treatments or irradiation are needed. Safe resection of as much tumor as possible and symptomatic control is aimed at, rather than remission. Many procedures are required for rapid progression of lesions or recurrences, in order to extend the survival of the patients. Metastases of pituitary carcinomas within the cranial cavity or spine can be attacked. Since they can occur anywhere in the brain or spinal canal they require the entire battery of neurosurgical approaches. Unfortunately, in this group of pituitary tumors, the complication rates are higher than in primary operations of enclosed adenomas. The respective techniques with their facilities and limitations are reviewed in this article.

## Introduction

**Fig. 1:**
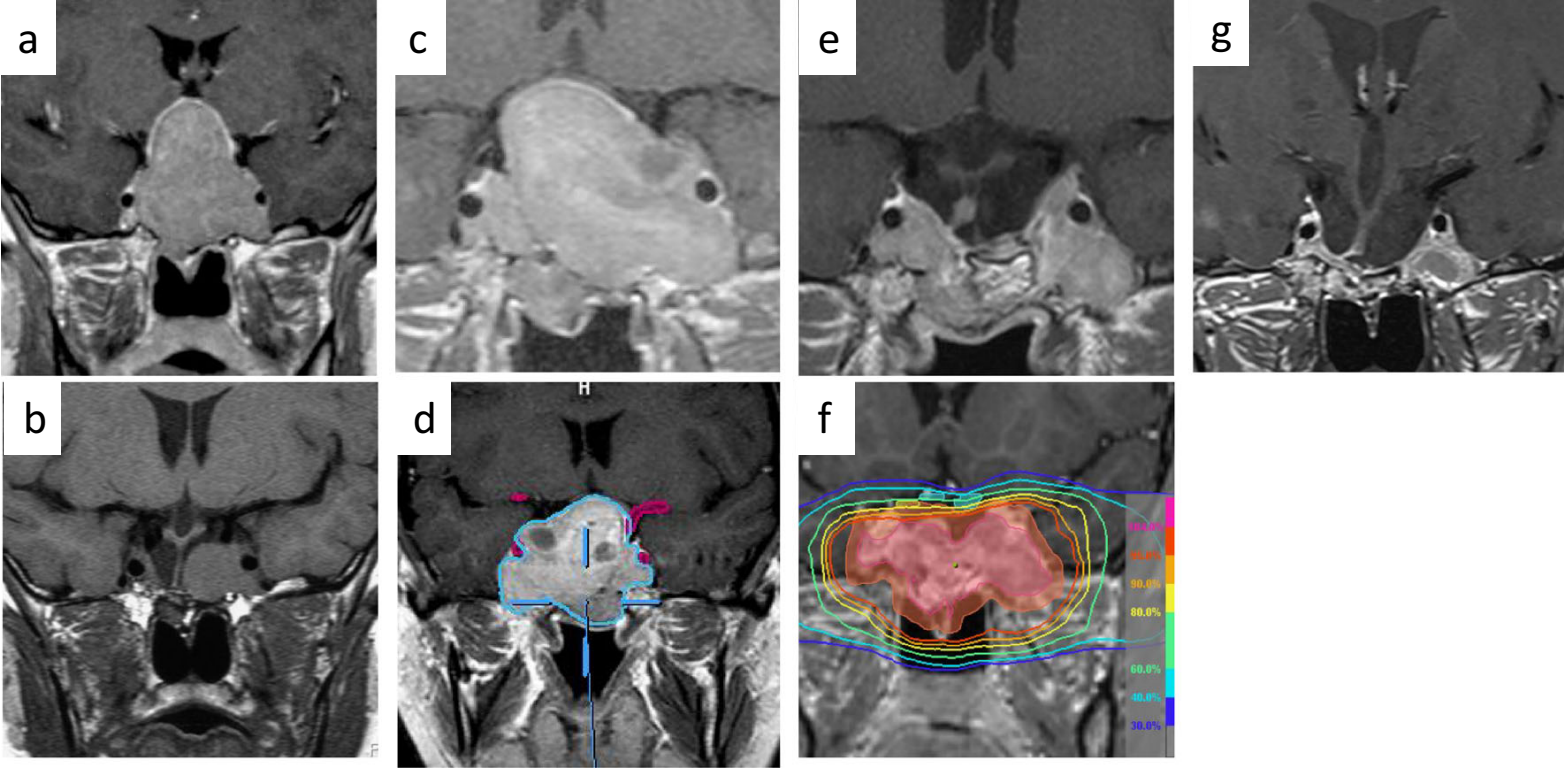
Rapid progression of a non-functioning pituitary adenoma in a 30-year-old female patient, as depicted on T1-weghted coronal MR images: **a** on presentation **b** delayed postoperative imaging 3 months after transsphenoidal surgery **c** follow-up investigation after 2 years **d** superimposing tumor structures onto operative field during repeat transsphenoidal surgery **e** delayed postoperative imaging after repeat surgery **f** RT-planning of residual tumor **g** most recent follow-up 5 years after conformal fractionated irradiation

Surgical treatment of pituitary adenomas usually aims at normalization of hormonal oversecretion in cases of secreting adenomas, recovery of visual function and total tumor resection. Aggressive pituitary adenomas are not well defined. However, most authors agree that they are characterized by invasive growth, rapid progression, resistance towards medical treatments and a tendency to recur [1, 2]. Thus, they are difficult to treat and the therapeutic efforts are mostly directed towards tumor control rather than surgical cure. Thus, frequently a combination of surgery, medical treatments, irradiation, and sometimes also chemotherapy are required [2, 3]. The entire therapeutic armamentarium is needed and interdisciplinary cooperation is mandatory, involving endocrinologiscts, neurosurgeons, radiotherapists and also oncologists [2]. Many operations in this context are not designed to excise all tumor completely but rather to reduce the tumor size in the best possible fashion, hopefully avoiding complications. With this utmost possible volume reduction, tumor size and extend of oversecretion are reduced and the outcomes for further treatments, radiotherapy and antiproliferative drugs, respectively, are more favourable. Size reduction lower radiation exposure of neighbourhood structures, such as visual pathways and brainstem. Thus, collateral damage from irradiation is lessened and medical antisecretory and antiproliferative treatments become more efficacious [4]. The concept of size reduction in a tumor that is not deemed to be complete resectable by an operation is called debulking. The standard surgical approaches have to be modified or combined to expose and resect tumor in the best possible fashion. Rapid progression of residual tumour or recurrence frequently requires reoperations (Fig. 1). The indication for repeat operative tumor resections sometimes determines the survival of the patients.

Pituitary carcinomas are defined by the presence of metastases. Resection of these requires the entire battery of cranial and spinal approaches, which are available in modern neurosurgery. During the progression of adenomas with rapid regrowth, repeat operations may become necessary to achieve symptomatic control, maintain mobility of the eyes and vision or control intracranial pressure. The neurosurgeon plays a pivotal role in the management of aggressive pituitary adenomas and pituitary carcinomas, and frequently coordinates the care of such patients with aggressive tumors within an interdisciplinary team.

## Preoperative investigations

**Fig. 2:**
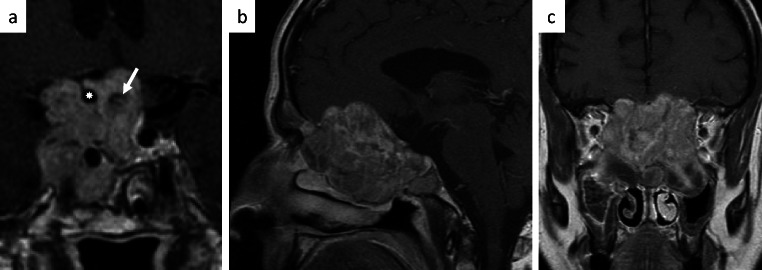
Invasive growth pattern of pituitary adenomas, encasing the internal carotid artery (✸) and optic nerve (arrow) (**a**) or growing and destroying along the anterior skull base and medial wall of the right orbit (**b** + **c**)

Preoperative planning and decision making about the optimal individualized strategy is usually based on preoperative imaging. Unless there are contraindications, to date a proper magnetic resonance image (MRI) dataset is mandatory. It reveals size, localization and extent of the lesion. In recurrent and progressive tumors, an analysis of the entire image set that reveals evolution of the tumor is recommended, since it allows to trace from where the tumor originated or where it predominantly progressed (Fig. 2). If needed an additional a CT-scan of the skull base helps to identify infiltration or destruction of bone. Of course, if medical antiproliferative treatments are available, such as in prolactinomas, the imaging series should include the initial situation and response to drug treatment. The goal of surgery should be determined in an interdisciplinary conference. Usually, resection of as much tumor as possible and thus reduction of the lesion to the minimal possible size is attempted. Visual function should be assessed preoperatively and pituitary function, both clinically and by laboratory investigations of the respective hormones. Moreover, hormonal oversecretion should be determined according to current guidelines. Sometimes in such tumors, one single surgical approach is not sufficient to attack different tumor portions. In these instances, the combination of transsphenoidal and transcranial operations has to be considered, initially, or with some delay, when the result of primary surgery is visible [5, 6].

## Transsphenoidal surgery

**Fig. 3:**
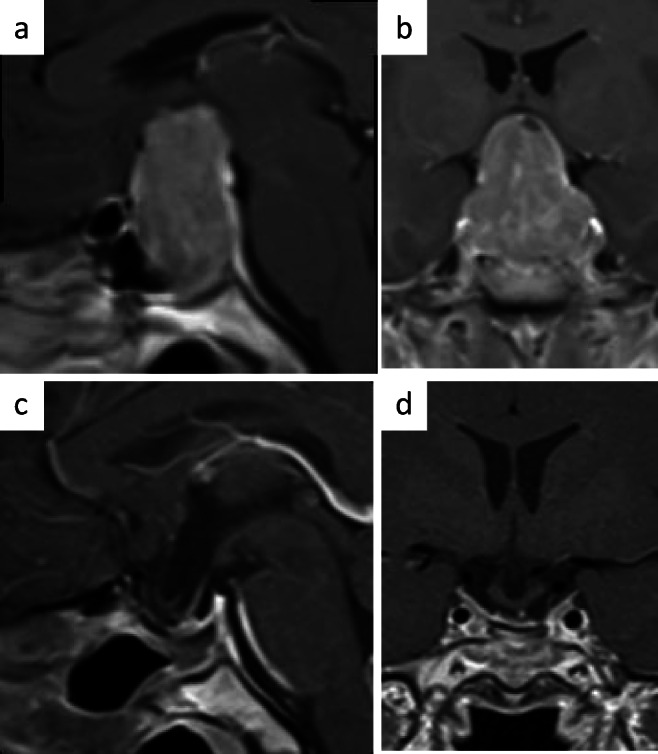
Giant size is not a limit for tumour resection as long as there is a sufficient communication between intra- and suprasellar components (**a**+**b**). Depite of the large size, the tumor could be readily resected (**c**+**d**) as the delayed postoperative MRI shows

The transsphenoidal route is to date the standard operation through which most pituitary tumors can be approached and resected. The operation has undergone refinements over more than a century. It evolved dramatically since the introduction of the operating microscope and the use of the roentgenologic image intensifier. The transsphenoidal approach has many variants, and includes direct perinasal, sublabial or paraseptal approaches with or without the use of a nasal speculum or dissection of the nasal mucosa [5, 7]. One disadvantage of the transsphenoidal approach is the deep and narrow working corridor. Thus, specific instruments are appreciated, which are particularly designed just for this kind of surgery. To date, there are basically two options for visualization, which also describe the two basic variants of the operation: One is the microsurgical technique for with which a nasal speculum is necessary. Alternatively, an endoscope can be used for visualization where light source and lens are introduced into the sphenoid sinus and into the tumor cavity, respectively. With both visualization techniques x-ray flouroscopy or or neuronavigation can be used, to ascertain proper orientation. Diamond drills are used to open skull base and sellar floor, particularly in patients with incompletely pneumatized sphenoid sinus. The sellar dura is identified and fenestrated. Tumor resection is performed by using curettes and microforceps from all portions of the tumor cavity. A generous exposure allows adequate access and the opportunity to identify and manipulate structures. In microadenomas, the normal gland is easily found and separated from the adenoma, whereas in large tumors, the normal pituitary is much more difficult to identify. However, it is always actively searched and preservation attempted. The usual intraoperative estimate of tumor resection in pituitary macroadenomas is the visualization and degree of descent of the the sellar diaphragm. This frequently consists only of arachnoid of the optico-chiasmatic cistern but might occasionally be covered by a thin layer of normal pituitary tissue. If the connection of intra- and suprasellar tumor portions is sufficiently wide, even giant adenomas can nicely be extracted via a rather standard transsphenoidal operation (Fig. 3). In extrasellar expanding tumors resection of the clivus or the anterior skull base might be needed to obtain the ability to resect such tumors completely. With the introduction of the endoscope those, the so called “extended” transsphenoidal approaches have been developed [8]. In aggressive tumors, the transsphenoidal approach needs thus to be modified to fit the requirements of exposure and dissection of the individual tumor. The additional use of the endoscope, whenever this deemed useful, is of course encouraged during microscopic transsphenoidal operations [9] unless anyway an entirely endoscopic operation was chose for the entire surgical procedure. There are reports of additional tumor tissue extractions during endoscope-assisted microsurgical operations [10]. When the most possible radical resection of the tumor has been finished, meticulous hemostasis is performed. One problem of the transsphenoidal approach is the closure of the osseous defect of the sellar floor, particularly if cerebrospinal fluid (CSF) leaks and especially in extended transsphenoidal approaches. Thus, there is a variety of techniques described for the reconstruction of the skull base, including gelfoam, autologous transplants, like fat, fascia, or bone, fibrin glue, direct suturing, the use of a pedicled muco-periosteal flap, and others [5, 7, 8].

## Transcranial surgery

**Fig. 4:**
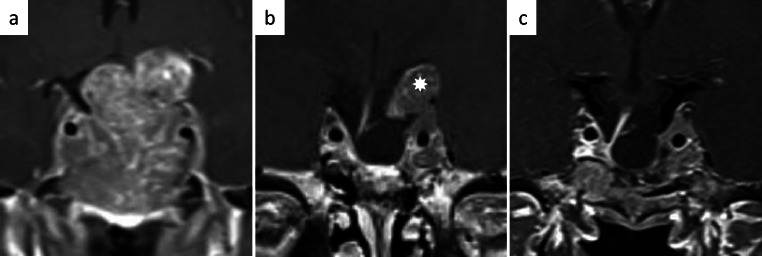
A 48-year-old female patient presented with a large and invasive adenoma and a 3rd nerve palsy of the left eye (**a**). After transspehnoidal debulking surgery a supraclinoidal parasellar tumour (✸) persisted (**b**), which latter was resected via a fronto-temporal craniotomy (**c**)

Transcranial approaches are still sometimes needed for tumors which are mainly localized outside of the sella or if the sella turcica is inadequately expanded in respect to the size of the lesion [11]. Standardized craniotomies are used, which are performed close to the skull base so that brain retraction is avoided. Depending on the localization of the tumor fronto-lateral, fronto-temporal (*pterional*) or basal midline craniotomies are performed. The drainage of cerebrospinal fluid and/ or resection of sphenoid bone usually creates sufficient space to microsurgically dissect the carotid artery and is branches as well as the optic nerves and chiasm, respectively. The arachnoidal planes within the optico-chiasmatic cistern are detached from the surface of the tumor. Usually, already at this stage an attempt is made to identify the infundibulum. Once the course of the major arteries of the anterior circulation is identified, the capsule of the tumor is incised and the lesion resected in a piecemeal fashion (Fig. 4). One must consider that the tumor capsule of the adenoma in huge lesions is the deformed, compressed and flattened pituitary gland. There are several corridors through which the tumor can be dissected: between the ipsilateral carotid artery and the optic nerve, between both optic nerves, lateral of the branches of the carotid artery and through the lamina terminalis. A careful dissection under direct vision and maintenance of anatomical cleavage planes seems crucial. The tumor may be traced posteriorly until the arteries of the posterior cerebral circulation are dissected. The authors prefer a pterional craniotomy for anteriorly located suprasellar lesions and a midline fronto-basal craniotomy with subsequent interhemispheric dissection for lesions that extend posteriorly to the clivus level. In the latter situation, the olfactory nerves need to be dissected and released from their arachnoidal sheets. In tumors with interventricular extension, paramedian or medial frontal craniotomies may be needed which allow transventricular exposure and resection of the tumor or a spatially restricted corpus callosotomy. The transcranial approaches allow a relative radical resection of the intracranial portion of a pituitary tumor. However, the major challenge is the risk of damage to the surrounding structures, which must be exposed and dissected such as blood vessels, the visual pathways, the infundibulum, pituitary gland and the hypothalamus [5, 7, 11, 12].

## Combinations of approaches

If adequate size reduction of an aggressive tumor cannot be achieved by one operative approach alone, a combination can be considered. For this purpose, simultaneous transsphenoidal and transcranial operations have been reported [13–15]. Virtually all combinations of approaches have been described in case reports of individual patients or in descriptions of small series, irrespective of microscopic or endoscopic operation techniques [6, 15]. The authors, however, prefer staged procedures [5], with either limited transsphenoidal resection of the basal portion of a huge tumor that has invaded brain and suprasellar arachnoid or with a craniotomy as the initial step (Fig. 4). A few weeks in between the procedures seems to be an adequate interval [5, 6]. How to proceed is a matter of individual decision depending on the characteristics of the tumor and the experience of the surgeon with the individual approaches.

## Invasion of the cavernous sinus

**Fig. 5:**
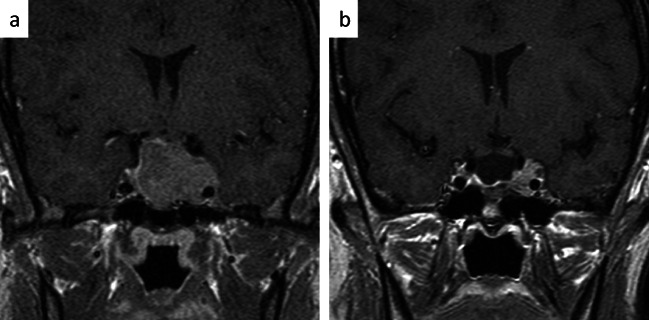
Limitations of transsphenidal tumor resection by cavernous sinus invasion: In this Knosp 4 grade tumour (**a**), the parasellar tumour portion could not be resected for the invasive nature of the adenoma, which has encased the intracavernous carotid artery (**b**)

Invasion of the cavernous sinus restricts very much the chance to resect a tumor completely. Usually, the parasellar tumor portions are traced along the preexisting communications which the adenoma has created [16, 17]. The extent of parasellar tumor growth and cavernous sinus involvement is appreciated from preoperative MRI by using Knosp scale [18] or its updated modification [19] which predicts the likelihood of invasion. A line drawn through the intracavernous portion of the carotid artery is used as an auxiliary line. Tumor lateral of the course of the carotid artery (Fig. 5) generally speaking is deemed not to be completety resectable [5, 20]. This has prognostic significance for the chance to totally resect a pituitary tumor and consequently also to achieve endocrine remission in hormonally active pituitary adenomas. However, debulking is, of course, possible, even if no aggressive techniques are applied and the tumor is only traced along the perforations which it has created during its expansion [16, 17, 21]. For minor involvement and even invasion of the medial wall of the cavernous sinus, deliberate resection of the medial wall has been proposed [22]. The authors strongly advocate against deliberate opening of the cavernous sinus via a transcranial approach for the damage to the optomotoric nerves and risk of lesion of the intracavernous carotid artery [23]. Vigorous attempts to resect invasive parasellar tumor via a transsphenoidal approach can also be dangerous [24]. One of the basic technical problems is that bleeding from the cavernous sinus can infrequently be controlled by coagulation. Only if both blades of the dural walls can be coagulated together, this works satisfactorily. In all other instances, the bleeding is rather increased if the laceration is coagulated. Gentle compression with gelfoam, however, works well and finally, the materials can be sealed with fibrin glue or fibrin coated gelfoam [5].

## Invasion of the sphenoid sinus

When the adenoma has inferiorly perforated the sella floor and the basal dura, usually the mucosa of the sphenoid sinus gets invaded. The sella floor is then radically resected with rongeurs or forcepses and the residuals drilled. The dura can be excised, at least between the walls of the cavernous sinus. Thereafter, the mucosa of the sphenoid sinus is detached from the bony walls of the sphenoid sinus and radically resected [21]. During this procedures, the ENT principles of resection of malignancies of the paranasal sinuses are applied, of course utilizing the endoscope to visualize the regions of interest as perfectly as possible [25]. Frequently, however, bleeding restricts visualization. Bleeding from invaded bone can be reduced with the diamond drill [26]. Multiple applications of hydrogen peroxide and tamponades are usually sufficient within the sphenoid sinus. With gross invasion, a total resection of the aggressive adenoma is technically impossible.

## Invasion of clivus and skullbase

Invasion of the upper clivus requires a lower posterior transsphenoidal approach. This is an application of extended transsphenoidal surgery. A useful landmark is the junction of posterior sellar floor and clivus. From there the clivus can be drilled and thus invaded skull base bone removed [21]. Exposure of the middle clivus is no more possible, even with an extended transsphenoidal approach [8] and consequently requires transoral surgery splitting the posterior pharynx, such as in surgery for clival chordoma [27] The clival dura serves as the most important landmark. Laterally, the carotid arteries pose some threat in this situation. Thus, the use of navigation is encouraged.

## Invasion of arachnoid and brain

Previously, one considered invasion of arachnoid and brain in pituitary adenomas with remarkable suprasellar extension which had no or very minor impairment of vision (Fig. 2a) [26]. To date, invasion is usually suspected from the MRI when irregular suprasellar confines and/or encasement of arteries of the cerebral arteries is directly depicted. While debulking of basal tumor portion can be accomplished via transsphenoidal approaches, the superior tumor portions with arachnoidal invasion are ideally dissected under direct vision via a craniotomy [11, 12]. The major arteries are exposed early during such operations so that vascular control is always possible by temporary clipping if required. Thereafter the tumor is gently dissected from the major blood vessels and arachnoidal sheets. It is general experience that minor vessels, which run in the invaded arachnoidal layers, will be sacrificed. Perforating arteries, however, must be preserved under all circumstances. Consequently, the resection will remain incomplete. Likewise, adenoma invading the brain, will be microsurgically dissected and extracted. The remaining tumor cavities with pial and arachnoidal defects are then coated cautiously with surgicel.

## Resection of cranial metastasis

**Fig. 6:**
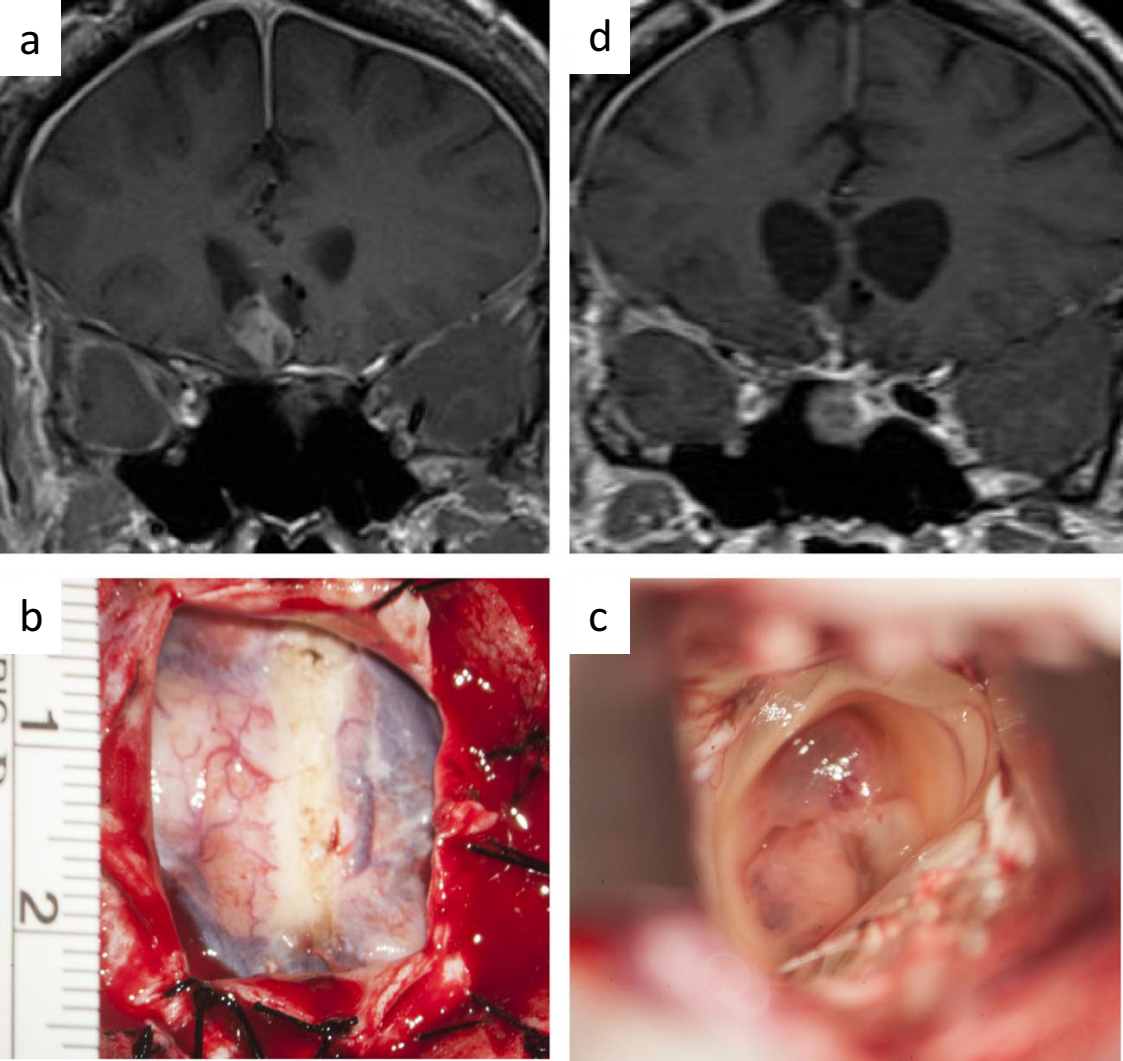
Progressive cerebral metastasis at the bottom of the right lateral ventricle of a PRL-secreting pituitary carcinoma (**a**). A small right frontal craniotomy (**b**) and transventricular approach (**c**) was chosen to resect the metastasis (**d**)

Once intracranial metastases within the anterior, middle of posterior cranial fossa are detected by MR imaging, they are usually directly attacked via appropriate craniotomies. For the resection of cranial metastases the respective, most suitable cranial approaches may be necessary, applying the principles of microsurgical brain tumor operations [28]. Ideally, the chosen approach allows for the shortest possible access to the lesion without the risk of additional neurological deficit. The support of navigation guidance is appreciated (Fig. 6).

## Resection of spinal metastasis

**Fig. 7:**
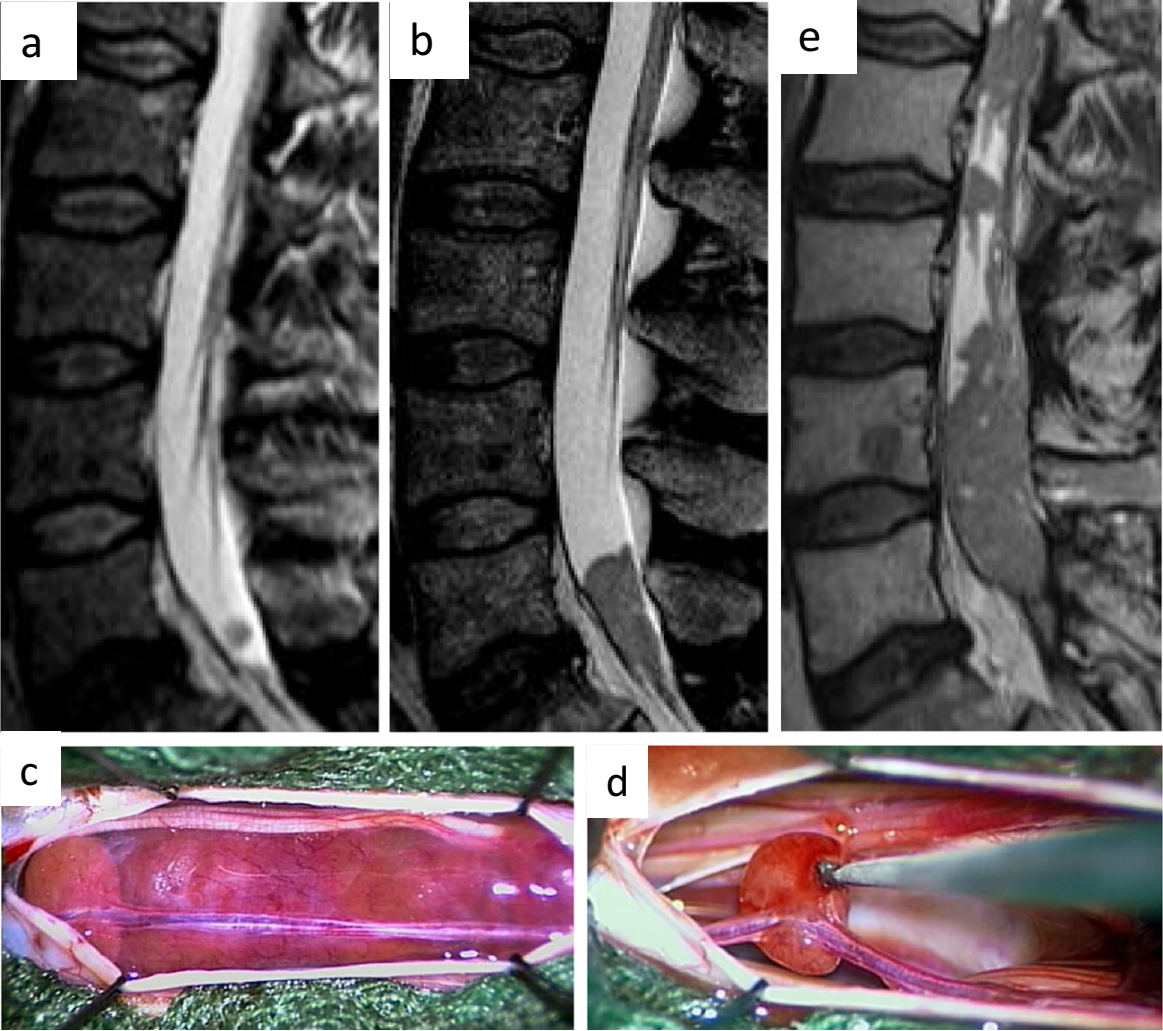
Progressive spinal metastasis of a Proalctin-secreting pituitary adenoma. After first appearance (**a**) the metastasis showed a rapid progression (**b**). Intraoperative views (**c** + **d**) of the adherent metastasis. Despite irradiation and medical therapy with dopamine-agonists and temozolomide the spinal metastasis progressed continuously (**e**)

Like intracranial metastases, spinal metastases can be exposed and resected to reduce total tumor volume. Usually, a cervical or lumbar laminectomy are required. The principles of surgical treatment of intradural spinal tumors are applied [29]. The size and extent of the metastases determines the surgical procedure (Fig. 7). Stability preserving approaches, such as hemilaminectomy or laminoplasty should be preferentially used. When nerve roots or the myelon are infiltrated, only a partial resection of the lesion is possible. However, there is no doubt that resectable tumor should be excised before adjuvant treatments are postoperatively administed [28].

## Technical supports

Any kind of technical support apart from the usual microsurgical equipment is appreciated in this kind of difficult to treat tumors. [9]. The laser Doppler system helps to avoid arterial vascular injury. Neuronavigation can provide information about the localization and extent of the tumor, the relationships to the anatomical landmarks and compensate loss of information if the latter ones are no more available since they have been resected in previous interventions. Intraoperative imaging can deliver intraoperative assessment on the extent of tumor resection and depict residual tumor [9].

## Results

The surgical results are measured by the amount of tumor size reduction, as documented by the delayed MRI or CT images, reduction of hormonal oversecretion, recovery of vision, and the percentage of total tumor resections. The rate of remission in tumors with lateral extension into the cavernous sinus or infiltration of the sphenoid sinus drops with tumor size and the magnitude of extrasellar extension. Jane et al. [30] demonstrated this very convincingly in patients with acromegaly who underwent endoscopic transsphenoidal operations by using the Knosp score [18, 19]. Micko et al. [20] likewise confirm, that endocrine remission can hardly ever be expected in tumors with major parasellar invasion. Once this is determined in the MRI, usually tumor within the cavernous sinus will remain, whatever technical modifications are used. However, the scales only predict a statistical likelihood of invasion and thus, it is always worthwhile to give it a try and attempt the most possible radical extraction of a parasellar lesion. Park et al. [31] report on remarkable remission rates in such tumors. Decompression of visual pathways can rescue vision even in giant pituitary adenomas [6, 13]. Diplopia, however, resolves reliably, irrespective if the parasellar adenomas are invasive or not, once an adequate decompression has been achieved [21, 20]. The dependency of surgical results on the size of the lesion and on infiltration of the sphenoid sinus were already described by Hardy [32] many years ago and confirmed in numerous more recent publications. Giant adenomas have a low remission rate of remission of hormonal oversecretion but even partial resection may improve the effect of postoperative medical treatments [33]. At least, this has been shown for tumors causing acromegaly by comparing the action of somatostatin analogs pre- and postoperatively [4].

## Complications

Although almost each and any complication of intracranial neurosurgery can theoretically occur, the most frequent complications of pituitary surgery are CSF-leaks, meningitis loss of vision, diplopia, major blood loss during surgery, rebleeding, hypopituitarism and diabetes insipidus [34–36]. They occur with variable frequency, depending on the patient cohort, the underlying endocrine disorder, size and invasion of the tumors and the fact whether primary surgery or reoperations are performed. The case load of the center and the experience of the surgeon are also key factors. Generally speaking, morbidity and mortality of pituitary surgery are much higher in aggressive, large and invasive tumors, as compare to enclosed macroadenomas. Particularly transcranial approaches to the cavernous sinus [23] extended dissections of parasellar tumor portions [24] and extractions of giant tumors are affected with major complications, far beyond that, what is expected in average series of patients with pituitary adenomas [36]. With spread of the disease into other compartments of the central nervous system, other complications of operative treatments are more likely than if the tumors are confined to the sellar region. It must be considered in the decision making if the amount of tumor that can be resected or the decompression of a structure is worth the additional risk.

## Summary

Surgery for aggressive pituitary adenomas is the operative, technical portion of an interdisciplinary challenge. Strategic planning and timing of surgical (re-) intervention have to be matched by postoperative medical and/ or radiotherapeutic treatments. Ideally, surgeons to treat such aggressive tumours have a broad experience of operative treatment of pituitary adenomas and skull base surgery to choose the most effective approach with maximal tumor resection and minimal risks for the patient. Transsphenoidal approaches and especially their extended variants offer a broad variety of possible accesses to an invasive pituitary tumor. Compared to non-aggressive pituitary adenomas transcranial approaches have to be used more frequently. In the presence of a pituitary carcinoma even more aggressive surgical treatment and the entire battery of potential neurosurgical approaches are necessary to lower the tumor burden prior to further treatment.
